# Evaluation of compliance and accuracy in Parkinson’s disease motor symptom tracking: a comparative study of digital and traditional paper diaries using a smartphone application (MyParkinson’s)

**DOI:** 10.3389/fneur.2025.1522721

**Published:** 2025-01-29

**Authors:** Nazli Durmaz Celik, Asli Yaman Kula, Naci Murat, Müge Kuzu Kumcu, Aydan Topal, Serhat Özkan

**Affiliations:** ^1^Department of Neurology, Eskişehir Osmangazi University, Faculty of Medicine, Eskişehir, Türkiye; ^2^Department of Neurology, Faculty of Medicine, Eskişehir Osmangazi University, Eskişehir, Türkiye; ^3^Department of Neurology, Bezmialem Vakif University Hospital, Istanbul, Türkiye; ^4^Department of Industrial Engineering, Ondokuz Mayıs University, Samsun, Türkiye; ^5^Department of Neurology, Lokman Hekim University, Ankara, Türkiye; ^6^Department of Neurology, Samsun Training and Research Hospital, Samsun, Türkiye

**Keywords:** mobile applications, Parkinson’s disease digital diary, smartphone application, motor symptom diary, Parkinson’s disease

## Abstract

**Background/objectives:**

This study aimed to evaluate compliance and accuracy in comparison with traditional PD diaries for tracking motor symptoms using a new smartphone application (MyParkinson’s) and paper diary strategies. Parkinson’s disease (PD) is a neurodegenerative disorder with progressive motor symptoms. Treatment becomes more challenging as PD progresses, motor complications in the form of wearing-off phenomenon and levodopa-induced dyskinesia develop. Traditional paper diaries and clinical scales used to evaluate patients may be inadequate in assessing whether the patient is “on” or “off,” resulting in less-than-ideal treatment changes.

**Methods:**

A randomized crossover design was utilized to examine 22 advanced PD patients who underwent symptomatic assessment with both diaries during two separate 24-h periods seven days apart. The compliance and accuracy of data were assessed by comparing diary entries with the clinical examination notes and WhatsApp queries. LaOerly, patients’ diary preferences were also evaluated.

**Results:**

The digital diary had significantly beOer compliance and accuracy than the paper diaries, with substantial/almost perfect levels of agreement (*κ* = 0.615 to 0.818) between logged symptoms and clinical examination notes. 65% of patients preferred the digital diary for follow-ups, and there was no significant difference in ease of use compared to paper diaries.

**Conclusion:**

Digital diaries are helpful in the clinical management of PD patients as they minimize recall bias and reduce data errors in appropriately selected patients. Our study suggests a broader adoption of digital health technologies in PD management. Still, additional research is necessary to improve the tools and assess long-term patient outcomes.

## 1 Introduction

Parkinson’s disease (PD) is the second most common neurodegenerative disorder in the elderly, affecting approximately 10 million individuals globally. It is characterized by a progressive loss of dopaminergic neurons in the substantia nigra, leading motor symptoms, such as bradykinesia, rigidity, rest tremor, and postural instability, as well as non-motor symptoms like sleep disturbances, orthostatic hypotension, and cognitive decline (1). Levodopa remains the cornerstone treatment, however as the disease progresses, patients often experience motor fluctuations, especially wearing-off and levodopa-induced dyskinesia, complicating treatment and affects quality of life (2). These fluctuations vary in intervals, weekly, daily, or even within the same day, but the patients are usually evaluated with history and clinical scales, as a snapshot examination. In this short time during clinical visits, patients may struggle to accurately recall their “on” and “off” periods, hindering optimal treatment (3, 4). Several paper-based patient diaries are specifically designed for patients with PD to help them track their motor symptoms (5). They are generally used in clinical practice for documenting patients’ motor complications or “on” and “off” periods within a specific time frame (e.g., hourly, every 30 min) for a day or a week in a structured manner. The most widely used paper diary for tracking motor symptoms was developed by Hauser et al. (6). Other paper diaries for PD have also been published (7–9). These diaries used to be a standard follow-up method, providing valuable insights into experiences and motor fluctuations through the time interval (data duplication), however, diary fatigue, filling in the retrospectively at once, and incomplete-missing-illegible diaries can undermine the quality of the data and potentially lead to suboptimal treatment decisions (10).

Thus, digital health technologies have introduced wireless electronic diaries (e-diaries) as a contemporary approach to monitoring PD symptoms and overcoming these challenges. Available on multiple platforms, including tablets, smartphone apps, and the web, these e-diaries have several advantages over paper diaries (11). Among them, smartphone app e-diaries stand out as they are convenient and easy to use. In response, these digital tools improve the timeliness of data entry (with alerts prompting patients to enter answers on time) and improve the accuracy of responses (5), which ensures data quality and helps improve patient compliance and retention (10). Although smartphone-based diaries hold promise for monitoring PD symptoms, only a few studies have been published to evaluate their use in monitoring PD symptoms. Since the adoption of these digital tools continue to develop, it becomes increasingly important to assess their utility.

Furthermore, the COVID-19 pandemic has considerably spread the use of digital health technologies to support the management of chronic conditions, for example, for patients with Parkinson’s disease (PD). In the last two years, many groups have emphasized the importance of using telemedicine and mobile tools in PD, focusing on their role in remote monitoring and individual management of patients with PD. For example, research by Dorsey et al. emphasizes the utility of telemedicine for providing care to PD patients during the pandemic (12). Moreover, wearable sensors have been used to provide accurate information about the continuous measurement of PD motor symptoms. Del Din et al. (13). A systematic review of wearable technology and its common uses in PD was performed, indicating that this technology can provide objective assessments of motor function and quality of life that can supplement traditional clinical evaluation. This suggests a paradigm shift in PD management, a new, more integrated, and technology-based era.

While these advancements are promising, the real-world application of digital tools to track daily PD symptoms is an area that has yet to be thoroughly investigated. This study aims to evaluate the effectiveness, compliance, accuracy, and patient preferences between traditional paper diaries and our new smartphone application (MyParkinson’s) in tracking motor symptoms in PD and evaluate the agreement between clinical examination notes and patient-reported data.

## 2 Materials and methods

### 2.1 Study design and participants

This single-center, prospective, randomized cross-over study enrolled 22 patients with advanced PD from June 2022 to January 2023. The UK Parkinson’s Disease Society Brain Bank criteria were used by one (14), and all participants who met these diagnostic criteria and had motor fluctuations and dyskinesias were enrolled in the study.

The study adhered to the principles of the Helsinki Declaration and received approval from the local Institutional Review Board (Approval No. 23/24.05.2022). Written informed consent was obtained from all participants before their inclusion in the study. Inclusion criteria were as follows: a baseline Mini-Mental State Examination (MMSE) score of at least 25 to ensure sufficient cognitive function, which could interfere with the results (15). For this study, we sought at least primary school graduates to enable comprehension of the instructions and effectively use mobile applications. In addition, Internet accessibility and smartphone use in this study were prerequisites because all motor symptom assessments of the patient were digitally monitored.

Power analysis was conducted using G*Power software (Version 3.1.9.7). Based on an expected effect size of d = 0.5, an alpha level of 0.05, and a desired power of 0.80, the analysis indicated that a minimum sample size of 20 participants per group was required SDF. The effect size assumption of 0.5 was based on findings from previous studies comparing digital and paper-based symptom-tracking methods, which reported moderate-to-strong differences in compliance and accuracy rates. This effect size was chosen to balance statistical power with the feasibility of recruiting participants for a resource-intensive crossover design study. By selecting this effect size, we aimed to ensure that the study was adequately powered to detect meaningful differences between the two diary methods while remaining feasible within the constraints of participant recruitment and study logistics.

Participants had to maintain a stable antiparkinsonian medication regimen for at least one month before and during the study to remove potential confounding effects possibly related to medication changes on the study outcomes. Individuals who took medications that might have altered their parkinsonism or dyskinesias and those with major depression, psychosis, or other severe medical conditions that could affect the results were excluded from the study.

All participants’ demographic and clinical characteristics, including age, gender, education, disease duration, motor complication duration, and Levodopa equivalent daily doses (LEDD) (16), were recorded. Motor symptoms of PD were assessed using the UPDRS Part III (17) and staged according to the Hoehn and Yahr (H&Y) scale (18). At the end of the study, patients were asked to evaluate both diaries in terms of convenience and preference.

### 2.2 Procedures

This study uses a randomized cross-over design to investigate compliance, accuracy, and patient preferences between traditional paper diaries and the electronic diary application MyParkinson’s for tracking motor symptoms in PD. To evaluate the reliability of digital and paper diaries, we compared hourly patient-reported entries from each diary with clinical assessments conducted during follow-up visits. A direct hour-by-hour comparison between digital and paper diaries was not performed to minimize the risk of compliance fatigue and potential data contamination. Requiring patients to maintain simultaneous records in both formats could have increased the cognitive and physical burden, particularly in a population with advanced Parkinson’s disease. Moreover, it might have introduced confounding due to copying between diaries. Instead, we used clinical assessments as the objective benchmark to evaluate agreement and reliability. This approach was intended to reduce recall bias and enabled an objective assessment of diary reliability without introducing additional biases from simultaneous dual recording.

### 2.3 Randomization and group assignment

The Participants were randomly assigned to begin with either the digital or paper diary, by Random. Org variations in the subjects. According to a computer-generated sequence of random numbers provided Random.Org (19). We used stratified randomization to balance across key demographic variables (age, sex, disease severity). Using this approach, participants with comparable characteristics were evenly distributed in their respective groups, reducing confounding factors. This method allowed us to have as much validity and reliability in the results as possible while assuming individual.

#### 2.3.1 Group 1

##### 2.3.1.1 First phase (Phase I)

*Day 1*: Participants in this group were initially given a paper diary. At home, they were instructed to record their PD-related symptoms (on–off fluctuations, dyskinesia, and tremors) by self-reporting every hour over 24 h to capture their motor fluctuations.

*Day 2*: First Clinic Visit: The day after the 24-h monitoring period at home, patients abended a clinic visit. During their follow-up visit, a movement disorders specialist evaluated their motor symptoms according to anamnestic data (interview in the medical routine without access to recorded diaries). We included this step to replicate the circumstance of a typical clinic evaluation, which is based on patient recall and clinician judgment.

##### 2.3.1.2 Second phase (Phase II)

*1 week later (Day 7-cross-over)*: Following a 1-week washout period to mediate any effects due to carry-over from Phase I, the PD patients were introduced, this time with access to MyParkinson’s digital diary. They downloaded apps on their smartphones and received standardized training in Turkish for using this system with a train-the-trainer model. They went on to track their motor symptoms every hour for 24 h.

*Day 8 –second clinic visit (cross-over)*: On the second day, motor symptoms were re-evaluated by the movement disorders specialist blindly (NDC, author) using a similar method to the first clinic visit to analyze and compare symptom data collected by paper diary vs. digital diary computed at home as a direct comparison of last measurement results from patient diaries.

#### 2.3.2 Group 2

##### 2.3.2.1 First phase (Phase I)

This group of participants started the study with a MyParkinson’s digital diary. They followed the exact same protocols as Group 1. They tracked their symptoms for 24 h, followed by a clinic visit the next day.

##### 2.3.2.2 Second phase (Phase II): (cross-over)

Like in Group 1, these participants switched to using the paper diary for another 24-h symptom-tracking after the one-week wash-out period. During a second clinic visit, their symptoms were then assessed blindly by the same movement disorders specialist (NDC, author).

All the patients were asked about their Parkinson’s-related symptoms via WhatsApp twice a day at randomized hours on both Day 1 and Day 7. These data were recorded and analyzed to identify discrepancies between what patients recorded in their diaries and what they reported in real-time for intra-rater reliability of each diary method.

### 2.4 Compliance and data analysis

Patient compliance with the diaries was assessed by comparing patient-reported diary entries with responses to randomized WhatsApp prompts sent during the study period.

Specifically, patients were prompted to describe their motor state at two randomly assigned times in diary-keeping days with a message. The diaries were then checked against real-time responses to highlight discrepancies and omissions. This strategy paralleled approaches from prior studies to confirm patient-report data as they occur in real time (20). The concept of real-time validation, indicating better reliability and compliance than traditional methods in their electronic data for Parkinson’s disease Additionally, real-time cues and alarms have been employed by others (8, 21) to improve patient compliance and lower recall bias, providing further validation for this approach. Patient compliance with the diaries was evaluated by retrospectively comparing the recorded status randomly asked via WhatsApp records at documented hours at the diary keeping days This analysis helped to identify discrepancies between what patients recorded in their diaries and what they reported in real-time, providing insight into the reliability of each diary method.

End of Study Evaluation Participants were asked to rate the paper and digital diaries in preference and convenience after the study. This charting is a subjective assessment that allowed us to estimate patient satisfaction as well as ease of usability.

**Figure 1:**
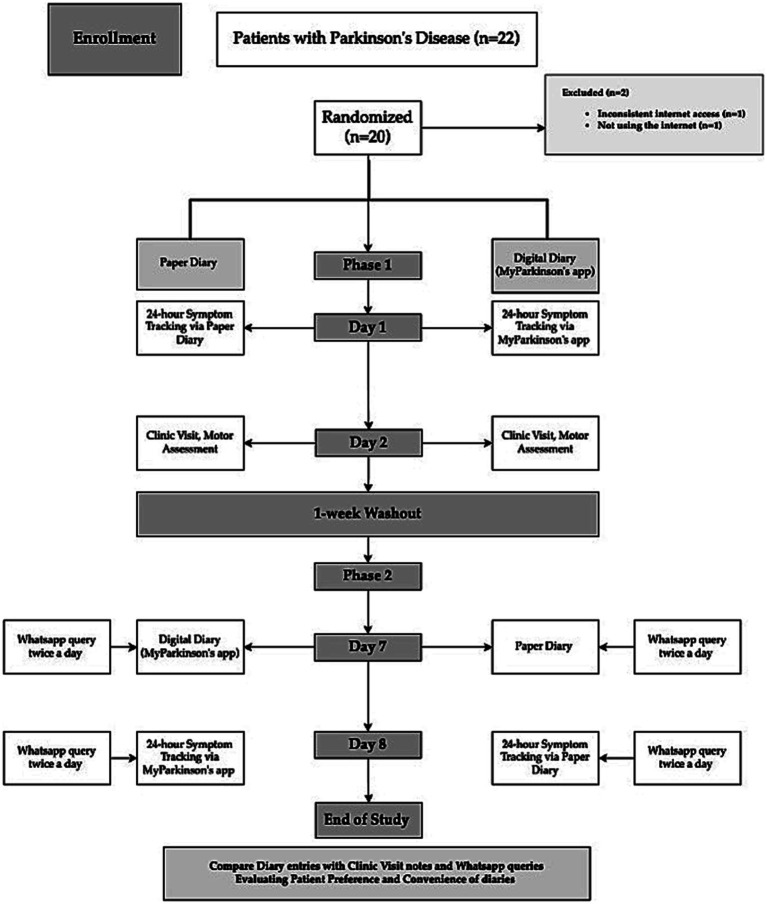
Flowchart of study design comparing paper diary and digital diary (MyParkinson’s) in Parkinson’s disease symptom tracking.

Randomization and Group Assignment are summarized in Figure 1.

### 2.5 Features of the paper diary

**Figure 2:**
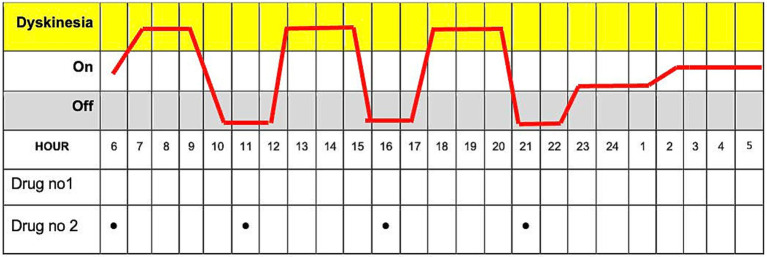
The paper diary is used to track Parkinson’s disease symptoms and medication usage over 24 h.

This paper chart is a 24-h diary for movement tracking designed explicitly for PD patients. With this diary, patients can systematically record and monitor their motor symptoms and medication usage (Figure 2). The diary is divided into hourly intervals between the starting point of 06:00 am and the ending point of 04:00 am (4,00 am of the following day), allowing for continuous observation throughout the day and night whenever the patient is awake. The essential columns in the chart are “On” and “Off,” which may indicate periods of the patient’s motor state. Columns such as “Tremor” and” Involuntary Movements” can track the most common symptoms of tremors and a side effect of dyskinesia caused by medication or a symptom of the disease. Finally, the record can be made in the column “Medication Use” to write which drug was taken at each hour. This Turkish chart is a revised form of Hauser et al.’s (6). This diary is more detailed to provide more understandable patient fluctuations daily and optimize treatment.

### 2.6 Features of the smartphone application digital diary (MyParkinson’s)

“MyParkinson’s” is a smartphone application developed by a movement disorders specialist (SO, author). The app is free to download from Google Play on Android and the app store on iOS. This novel digital motor tracking app for patients with PD provides a real-time, remote monitoring and quantification tool empowering physicians to optimize care. The app is like a diary that allows users to enter information about their motor condition, appear “On” and “Off” throughout the day, and involuntary movements such as tremors or dyskinesia typically occur in PD.

**Figure 3:**
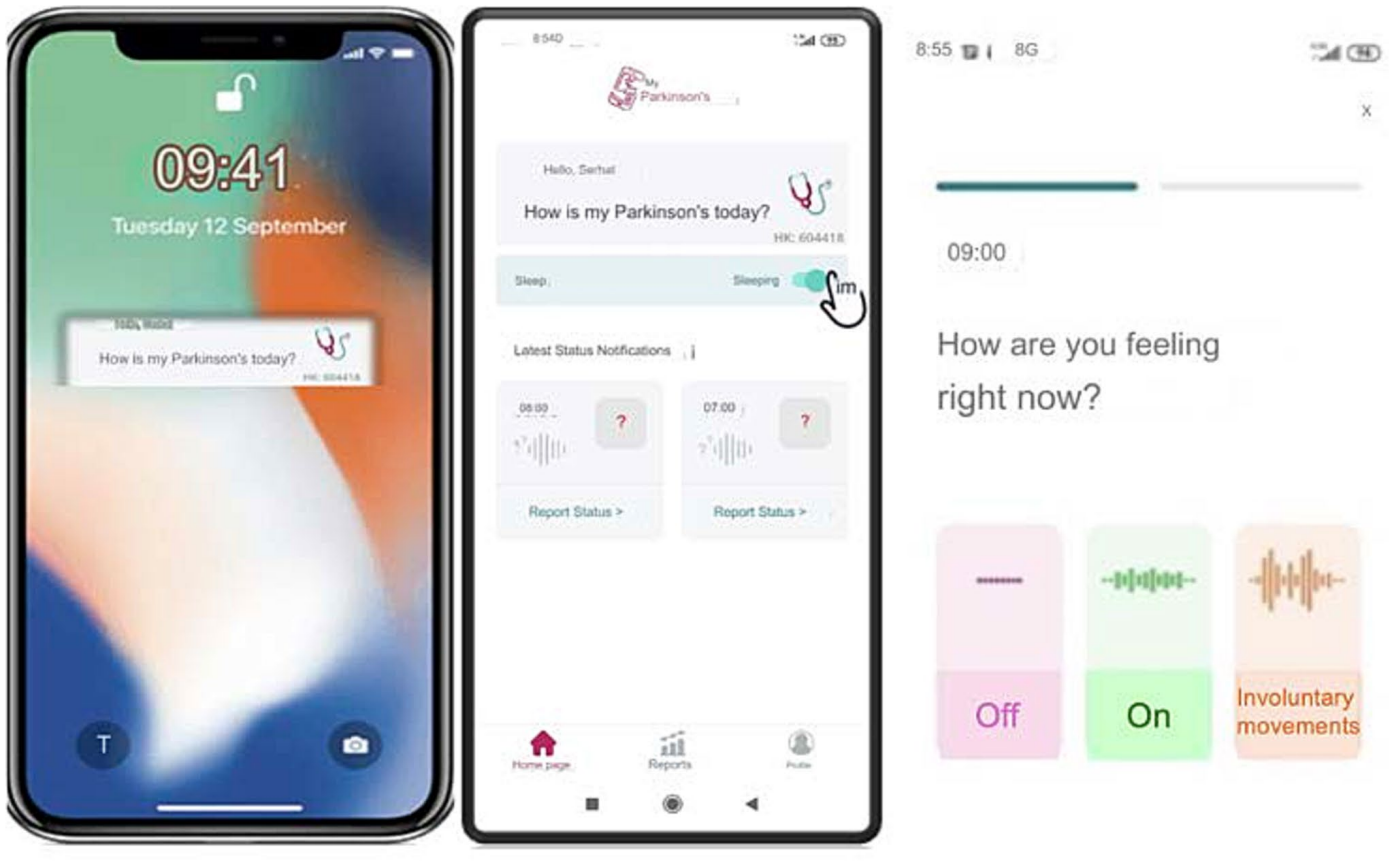
User interface of the ‘MyParkinson’s app for real-time symptom tracking and status reporting in Parkinson’s disease.

The app’s interface is designed with simplicity and accessibility, especially to make it easy for users to input their symptoms with just a few taps, considering it will be used mainly by older people. Once patients register in the app’s patient section, they will receive an ID on their screen. They should then share this ID with their clinician so the clinician can access the patient’s data in real-time using this code. The question ‘How is my Parkinson’s today?” pops up on the phone screen every hour or at the desired interval (from 15 min to every four hours) as a reminder. When they tap the question button, the patient is directed to the application and a page where the patient is prompted with the question, “How are you feeling now?” and is presented with three options: “off,” “on” and “involuntary movements” (Figure 3). This functionality enables real-time tracking, allowing the clinician to monitor the patient’s condition continuously. The data on the server can be accessed only by using an access code (handed to a physician by his patient), and this access does not work forever, as each report has its unique one-time code. This information is immediately shared with their clinician, with whom they have previously shared a unique patient code.

**Figure 4:**
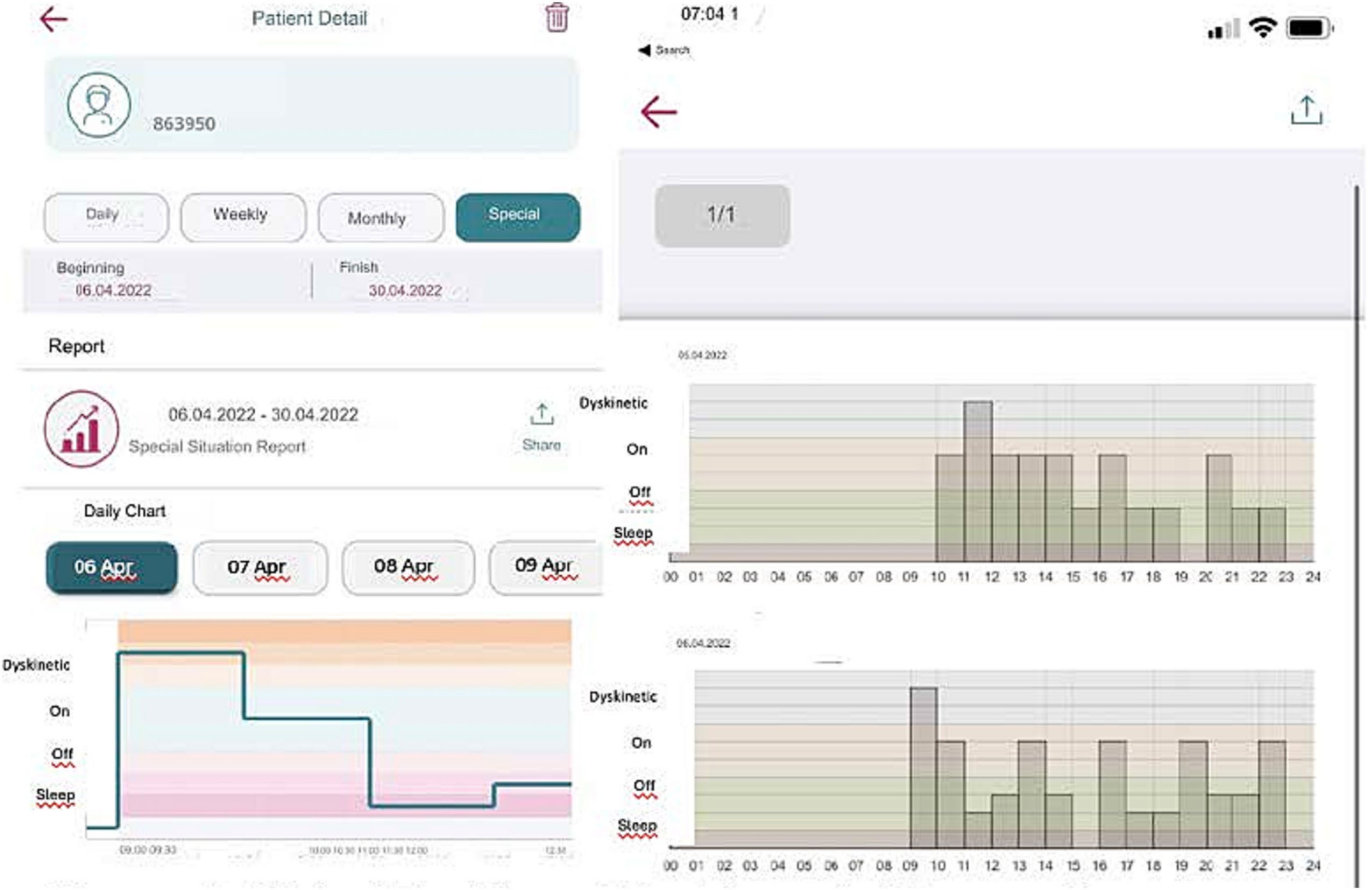
Digital tracking of Parkinson’s disease symptoms: 24-h report and dyskinesia monitoring of the ‘MyParkinson’s’ app.

If the patient forgets to answer or cannot answer the question, they can only respond to the next question. They are not allowed to go back and answer the previous question/s to ensure the integrity of the tracking system and prevent retrospective adjustments, changes in current data, or multiple entries at once. The app collects these entries into a single timeline and visually organizes them chronologically over numerous days. It then reviews detailed graphs displaying data over various intervals and adjusts treatments as needed (Figure 4). This feature has been added to be useful for patients and clinicians in determining when exactly symptoms are getting worse or when medication works best.

### 2.7 24-hour Parkinson’s disease motor symptom monitoring chart

**Figure 5:**
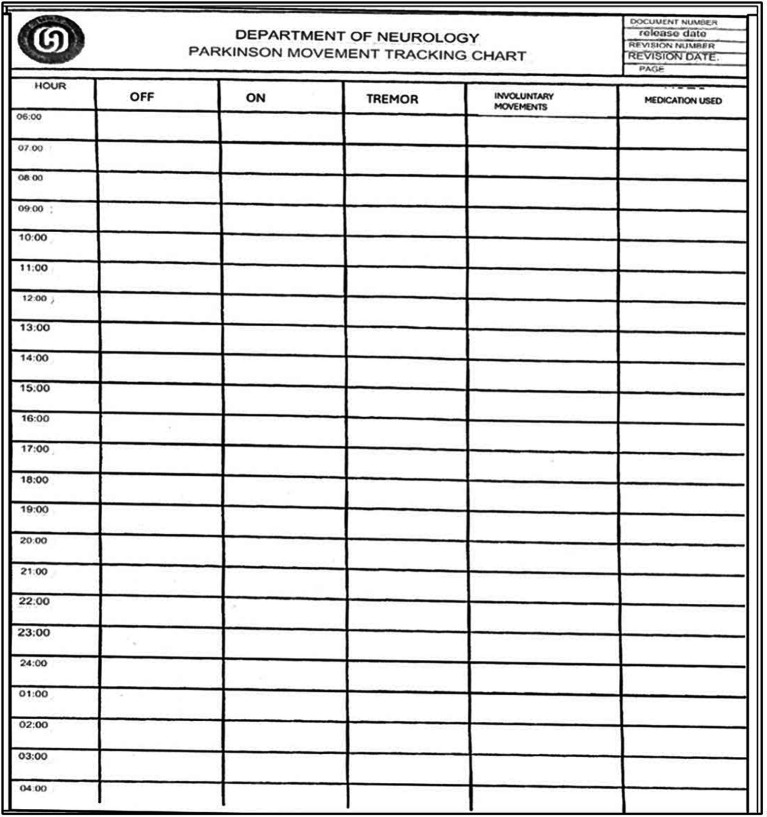
24-h Parkinson’s disease motor symptom monitoring chart.

This chart is designed for clinic visits in our department. During the visit, the clinician records motor fluctuations in dyskinesias and “on” and” off” periods based on the patient’s anamnesis. The timeline at the top of the chart spans from 6:00 am to 5:00 am the following day, with annotations marking the patient’s motor status at different times. Medication names are noted along the left side, with an arrow indicating when the patient took each medication. The chart visually represents the correlation between medication administration and the patient’s motor fluctuations, providing valuable data for clinicians to adjust treatment plans. The precise identification of” on” and” off” periods, along with times of dyskinesia, allows for a more tailored and effective management of Parkinson’s disease symptoms (Figure 5).

### 2.8 Statistical analyses

All statistical analyses were conducted using SPSS software, version 22. Descriptive statistics were presented as frequencies (*n*) and percentages (%) for categorical variables and as medians with corresponding minimum and maximum values for continuous variables. The weighted Kappa (*κ*) statistic assessed inter-rater agreement for each bias risk assessment tool domain and the overall evaluation. A medium effect size (0.5) was indicated based on previous effect sizes that have shown adherence and patient-reported outcomes to be medium-sized across studies comparing digital and traditional paper diaries in chronic diseases (22). We expect moderate group differences that metaphysically assume the significance of medium effects (effect size = 0.5), as Cohen suggested, which implies substantial differences between groups (23).

## 3 Results

This section may be divided by subheadings. It should provide a concise and precise description of the experimental results, their interpretation, and the experimental conclusions that can be drawn.

### 3.1 Demographic and clinical characteristics of the study cohort

Twenty-two patients were enrolled in the study; however, one patient was excluded due to inconsistent internet access, and another was excluded for not using the internet daily during the study period. Of the last cohort of 20 patients, 60% were female (*n* = 12). The mean age was 56.8 ± 10.2 years (range 34–70), and the mean Hoehn and Yahr (H&Y) score was 2.95 ± 0.65 (range 2–4). The mean levodopa equivalent daily dose (LEDD) was 1017.05 ± 461.5 mg (range 275–1997 mg), and the mean educational level was 9.15 ± 4.2 years (range 5–15 years). The mean duration of motor complications was 6.3 ± 3.06 years (range 3–6 years). The mean Mini-Mental Status Examination score was 27.8 ± 1.9 (range 25–30). No statistically significant differences were observed between the two groups with respect to age (*p* = 0.07), LEDD (*p* = 0.08), sex (*p* = 0.20), educational status (*p* = 0.22), or disease duration (*p* = 0.10).

### 3.2 Comparative analysis of paper and digital diaries in Parkinson’s disease management

#### 3.2.1 Patient enrollment and baseline characteristics

To measure compliance and agreement with clinical examination notes with diary entries, we use paper diaries compared to a smartphone application (MyParkinson’s) with a Turkish interface. The results of the patients who were first given paper diaries and those who began with a digital diary are shown separately. Patients who received a paper diary first were compared to those with their second phase (MyParkinson’s app stage) compliance characteristics and agreement between clinical examination notes, separately for each outcome. A detailed description of the agreement (Cohen’s kappa) between clinical examination notes and diary entries in patients initially receiving paper diaries is shown in Table 1. A comparison of patients starting with the paper diary showed a significant discrepancy in compliance; poor adherence occurred more frequently in the paper diary group versus the digital diary (MyParkinson’s) group. In the paper diary group, 37.5% of entries had no agreement; this percentage was only 6.25 in the digital diary arm. The digital diary had substantial and almost perfect compliance rates of 18.75 and 12.5%, respectively, compared to paper, which remains the lowest in the tier of substantial compliance (6.25%). In all 16 hourly timings, the digital diary had a better crude agreement with clinical examination than paper diaries (Table 2). We used Cohen’s kappa to evaluate the agreement between diary entries and clinical assessments. During measurements 2,3 and 16, the digital diary showed substantial to almost perfect agreement (*κ* = 0.615, *κ* = 0.818, *κ* = 815, respectively); however, for these periods, this was not observed using only paper diaries independently, no /slight agreement existed. This firm trend illustrates the true nature of digital diaries, which capture patient data accurately and reliably over time.

**Table 1:** Distribution of agreement between clinical examination notes and both diaries in patients who were first given paper diaries.

	*n*	%
Paper		
No agreement	6	37.5
Slight	2	12.5
Fair	4	25
Moderate	3	18.75
Substantial	1	6.25
Almost perfect	0	0
Application		
No agreement	1	6.25
Slight	4	25
Fair	4	25
Moderate	2	12.5
Substantial	3	18.75
Almost perfect	2	12.5

*The agreement was measured via Cohen’s kappa.

**Table 2:** The hourly comparison of the compliance characteristics of the clinical examination notes is a table.

	Paper diary (K)*	Digital diary (K)*	Paper diary	Digital diary
Measurement 1	0.574	0.483	Moderate	Moderate
Measurement 2	0.000	0.615	Slight	Substantial
Measurement 3	−0.071	0.818	No agreement	Almost Perfect
Measurement 4	0.524	0.259	Moderate	Fair
Measurement 5	−0.042	−0.143	No agreement	No agreement
Measurement 6	0.661	0.556	Substantial	Moderate
Measurement 7	0.500	0.623	Moderate	Substantial
Measurement 8	0.091	0.091	Slight	Slight
Measurement 9	0.000	0.388	Slight	Fair
Measurement 10	0.388	0.423	Fair	Moderate
Measurement 11	−0.111	0.783	No agreement	Substantial
Measurement 12	−0.111	0.123	No agreement	Slight
Measurement 13	−0.071	0.545	No agreement	Moderate
Measurement 14	0.583	0.388	Moderate	Fair
Measurement 15	−0.111	0.231	No agreement	Fair
Measurement 16	0.286	0.815	Fair	Almost Perfect

*K: Cohen’s Kappa test, Table column presents the corresponding data for the row.

In contrast, for the patients who received digital diaries first (Table 3), paper diaries continued to maintain significantly lower rates, even if less pronounced than those with paper diaries up front. However, the compliance shown with digital diary, in this case, presents a muddy picture showcasing poor overall compliance (25% entries). Significant proportions are compliant to different extents - 50% with slight and 18.75% with fir adherence.

**Table 3:** Distribution of agreement between clinical examination notes and both diaries in patients who were first given digital paper.

	*n*	%
Paper		
No agreement	2	12.5
Slight	3	18.75
Fair	6	37.5
Moderate	5	31.25
Substantial	0	0
Almost perfect	0	0
Application		
No agreement	4	25
Slight	8	50
Fair	3	18.75
Moderate	1	6.25
Substantial	0	0
Almost perfect	0	0

^*^The agreement was measured via Cohen’s kappa.

#### 3.2.2 Detailed evaluation of compliance across diary formats

The hourly comparison in Table 4 reflected that, when first implemented, the digital diary did not agree well with measurements. At this time, the overall agreement level of digital compared to paper print diary was still more vigorous in most cases for the original work.

**Table 4:** Hourly comparison of the compliance characteristics of the clinical examination notes and 16-hourly measurements of both diaries in patients who were first given a digital diary.

	Paper diary (K)*	Digital diary (K)*	Paper diary	Digital diary
Measurement 1	0.265	0.136	Fair	Slight
Measurement 2	0.524	0.000	Middle	Slight
Measurement 3	0.107	0.206	Slight	Fair
Measurement 4	0.524	−0.212	Middle	No agreement
Measurement 5	0.123	0.000	Slight	Slight
Measurement 6	0.219	0.000	Fair	Slight
Measurement 7	−0.111	0.184	No agreement	Slight
Measurement 8	0.206	−0.029	Fair	No agreement
Measurement 9	0.268	0.153	Fair	Slight
Measurement 10	0.474	0.231	Middle	Fair
Measurement 11	0.545	0.492	Middle	Middle
Measurement 12	−0.029	0.032	No agreement	Slight
Measurement 13	0.231	0.038	Fair	Slight
Measurement 14	0.000	−0.296	Slight	No agreement
Measurement 15	0.250	−0.167	Fair	No agreement
Measurement 16	0.464	0.231	Middle	Fair

*K: Cohen’s Kappa test, Table column presents the corresponding data for the row.

#### 3.2.3 User preferences and diary usability

Regarding diary preference, 65% (*n* = 13) of patients preferred to use the digital diary for future follow-ups, most often due to simple accessibility. In contrast, challenges, including lack of understanding (*n* = 2), limited internet access (*n* = 1), and functionality issues of the digital diary, were recognized as limitations for participants using this app. On the other hand, negative experiences of using paper diaries were tiredness throughout the day to complete (*n* = 4), difficulty carrying a pen and scrap of paper around during study visits (*n* = 3) and forgetting to fill the diary each hour. and keeping up to date with the diary on time, reported by six participants. Regarding the ease-of-use scale, there was no significant difference between the two diaries (*p* = 0.430). Subgroup analyses were also done to examine the effect of education and demographic factors on compliance and diary choice. Participants strongly preferred the digital diary (*n* = 5) when compared by education level, indicating its popularity for individuals with high school or university education. Participants with primary or secondary education (in contrast) demonstrated no preference, equally favoring both the digital app and the paper diaries. Such findings are consistent with earlier investigations indicating that digital literacy, typically correlated with educational level, plays a role in adopting and using digital tools (24). Furthermore, there were no notable differences in diary preferences by gender, indicating that preferences were more influenced by educational background than other demographics. However, we employed a relatively small sample size (*n* = 20), limiting the generalizability of subgroup analyses. More prominent studies are needed to confirm these trends and to understand better how demographics contribute to diary compliance and preference.

### 3.3 Clinical implications of results

The *p*-values were significant, indicating greater adherence to the digital diary than the standard paper diary. This translates to more accurate and timely tracking of symptoms that may facilitate targeted interventions in Parkinson’s disease (PD) management. The digital diary supplies trustworthy, real-time data that reduces the dependency on a patient’s wherewithal and can improve the accuracy of clinical decisions. Such conclusions help inform the clinical implications of our findings that demonstrate digital diaries and devices enhance the capture of patient-reported outcome measures compared with paper-based diaries. The importance of this enumeration lies in its potential for supporting personalized medicine by providing accurate and patient-specific data that can directly guide individualized therapeutic approaches. Schleidgen et al. reported customized medicine, by its nature, necessitates detailed and precise data to optimize clinical outcomes (25). The better engagement of patients in their treatment and follow-up observed through a new electronic tool, as reported here, could be translated into a possible better outcome and more effective treatment, as witnessed in this study, raising a link between statistics and clinical practice.

## 4 Discussion

This study aimed to assess the compliance and accuracy of a digital diary, MyParkinson’s, compared to paper diaries for tracking motor symptoms in patients with Parkinson’s disease (PD). The results showed considerable benefits of the digital diary over the conventional symptom tracking approach: enhanced compliance, more accurate data provision, and better usability, confirming PD management-related bottlenecks that a digital diary could solve. These findings align with global trends in digital health, highlighting the increasing integration of technology in enhancing patient engagement and clinical decision-making.

The COVID-19 pandemic has catalyzed the use of telemedicine and digital tools —even for Parkinson’s disease (PD) management. Marxreiter et al. found 75% of PD patients use the internet for disease information use (26), also instruments such as eDiaries (27) and the Parkinson’s Tracker App (28) enhanced medication adherence, symptom tracking, and patient engagement. Studies. demonstrated better compliance with digital diaries and finding of subtle fluctuations compared to paper diaries, although they found slightly lower compliance rates in some cases (9, 20, 29). Despite hurdles such as age differences and varying levels of technology familiarity, educating patients and designing more user-friendly interfaces can improve adherence. These use cases demonstrate the considerable promise of digital solutions for real-time monitoring and personalized treatment in patients with PD. While barriers like age and technology familiarity exist, patient education and user-friendly designs can enhance adherence, highlighting the transformative potential of digital tools for real-time self-monitoring and personalized care in PD. Digital tools have the potential to transform PD care by providing real-time self-monitoring and personalized treatment, though usability and patient support remain critical. Through the real-world approach of this study, comparing mobile applications to paper diaries, valuable insights into the practicality and clinical utility of digital tools in PD care have been obtained.

### 4.1 Superior compliance and accuracy of digital diaries

MyParkinson’s digital diary demonstrated substantial to almost perfect agreement with clinical evaluations (*κ* = 0.615–0.818), signifying its reliability for tracking PD symptoms. These findings corroborate previously conducted research, such as that done by Lyons et al. (21), which reported 99.98% near-perfect compliance rates with digital diaries relying on automated reminders to record symptoms in real-time. Similarly, Chuapakdee et al. (20) showed a high accuracy rate of 81.1% and user satisfaction with electronic symptom-tracking tools for PD, reinforcing the usefulness of digital platforms. Traditional paper diaries demonstrated significantly lower compliance and agreement with clinical data in our study, with 37.5% of entries showing no concordance compared to only 6.25% for the digital diary. These results are consistent with those of Löhle et al. (30), who found a modest overall agreement of only 59.8% (*κ* = 0.387) between assessments made in paper diary entries and clinical observations, and that paper-based tools struggle to capture the dynamic fluctuations inherent to motor symptoms. This paper argues that paper diaries often suffer from recall bias and incomplete entries, compromising data quality and limiting clinical utility.

### 4.2 Innovative features of MyParkinson’s

The superior compliance rates observed with MyParkinson’s can be attributed to its innovative features, which address common challenges associated with symptom tracking. Through real-time logging of symptoms, automated reminders help mitigate recall bias which can correlate data entries with motor states. This situation is critical in PD, where motor fluctuations vary widely throughout the day. Finally, MyParkinson’s does not permit entry backward in time, thus further increasing data accuracy by eliminating retrospective entry where the likelihood of making mistakes is high.

The app’s user-friendly design, tailored for older adults with cognitive and motor impairments, also helped achieve high compliance rates. Other digital tools like mPower (31) and Parkinson’s mHealth (27) also encompass self-management and research–while MyParkinson’s is primarily centered around real-time clinician access, allowing users to share data with physicians and initiate interventions (with alerts for scheduled visits). Not only does this feature help engage the patient, but it also allows for personalized and adaptive care, an area in which precision medicine excels. MyParkinson’s provides a localized interface (e.g., in Turkish) that differs from many international apps due to local language usage that is often neglected (as referenced in the Supplementary Table S1). Being free to use only opens the door for more potential, especially to be applicable in resource-constrained situations.

### 4.3 Comparison with other digital tools

The Supplementary Table S2 compares MyParkinson’s with other leading digital applications. On the other hand, apps like uMotif (28) and Roche PD Mobile Application V2 (32) track motor and non-motor symptoms, and MyParkinson’s currently focuses on motor symptoms. Although its specialization guarantees exact and strict monitoring of motor regimes, extending its features to regard non-motor states, such alterations of mood and cognitional aging, as well as depressions of slumber, would raise both the clinical usefulness of measuring and extend the vision utilizing evolution of PD to classic PD view. Moreover, a few advanced applications, like Roche PD Mobile Application V2, utilize machine learning algorithms to analyze symptom patterns and offer predictive insights. Completing the MyParkinson’s with similar analytic features could increase the app’s utility by helping clinicians predict symptom fluctuations and plan treatment strategies proactively.

However, MyParkinson’s remains the winner regarding simplicity, accessibility, and high-quality data. Its incorporation of automatic reminders and real-time data sharing makes it particularly applicable to clinical settings, whereby accurate and timely symptom tracking is imperative. Digital diaries such as MyParkinson’s are game changers in managing PD, allowing precise, real-time recording of symptoms. Ossig et al. (9) do a great job of pointing out how the integration of such tools with telemedicine and wearable sensors could augment remote care. However, older adults are less likely to be digitally literate, which can be challenging. Heart and Kalderon (24) suggested that using simplified interfaces, conducting training sessions, and involving caregivers can overcome such barriers.

We recorded over 24 h for our study, which provided important preliminary insights but did not assess long-term feasibility. Studies with longer study times over weeks or months to observe compliance might be ignored because of diary fatigue, as referred to by Stone et al. (22). Furthermore, integrating digital diaries with wearable sensors, as shown by Del Din et al. (13) may offer the potential for continuous, objective symptom monitoring, thereby minimizing participant burden and increasing reliability. These multimodal approaches are in line with emerging trends in digital health.

### 4.4 Limitations of the study

The main limitation of this study is that no direct hour-by-hour comparison of digital and paper diaries was done. Although that approach might yield deeper insights, participant fatigue, and deteriorating data quality are risks. Future studies should also be designed to facilitate concurrent comparison by using automated wearables or passive monitoring systems.

Also, their 24-h recording period was focused on reducing carryover effects. However, this may limit their ability to detect the full variability of motor symptomatology throughout the day, which is influenced by time-of-day factors such as the timing of medication and daily activities. Longer recordings are required to better understand fluctuations of symptoms across several days. Longer periods, though, could lead to less compliance due to participant fatigue and potentially disrupt daily routines, especially in older patients. To overcome these challenges, wearable sensors may offer continuous data with minimal burden, improving the feasibility of studies and data quality.

The small sample size also limits the generalizability of the study’s findings and diminishes the robustness of subgroup analyses. While our findings point to demographic differences concerning compliance, including education level, larger studies are needed for confirmation. Additionally, using digital diaries may be associated with a novelty effect leading to high compliance rates where participants were willing to comply with the protocol simply to benefit from the measurement of the new technology. While this effect is positive in the short term, it does not consider long-term engagement involvement in habitual performance.

The dependencies on self-reported data by patients, albeit limited by real-time logging, present a potential source of biases, especially among patients with cognitive deficits. Read more: Digital diaries and objective metrics from wearable sensors could complement each other for continuous, unbiased motor symptom monitoring: Changing the way to accurately assess parkinsonism. Studies like Ossig et al. (9) showed the efficiency of sensor-based technologies in characterizing motor fluctuations, which indicates that integration with the digital tool may also improve the reliability of the data.

### 4.5 Future directions

Future research in the healthcare setting must examine the duration of compliance and the applicability of digital diaries across demographics to address these limitations. Capturing both motor and non-motor symptoms using mobile phone applications such as MyParkinson’s would allow for a better understanding of the overall effects of PD, as many of these non-motor features affect the quality of life and indicate disease progression better than traditional assessments.

Digital diaries could also benefit from incorporating more sophisticated analytic features, including machine learning algorithms, to improve their ability to predict patterns or correlations. By learning from trends in symptom data, these algorithms might offer clinicians actionable information, leading to more proactive and targeted care. These advances would align with precision medicine’s objectives, which prioritize individualized treatment plans driven by consistent and precise patient data. Finally, the international interest in MyParkinson’s to meet global demand highlights the compelling case for developing multilingual interfaces and further localization of the app to cater to the worldwide audience and address the crucial gap in cultural adaptations of the digital health tools readily available for the community.

## 5 Conclusion

Our study strongly supports that a smartphone-based digital diary is adequate compared to traditional paper in terms of compliance and data accuracy of motor symptom recording for Parkinson’s Disease (PD). Digital diaries allow immediate data collection, decrease recall bias, and enhance patient involvement, making them an essential part of the multidisciplinary team managing PD. These benefits are core to broader trends in the shift toward digital health solutions for chronic disease care, mapping a pathway that eventually leads to personalized and proactive management for patients with PD. User-friendly digital technologies have a considerable potential to boost patient adherence and symptom-tracking accuracy, potentially revolutionizing Parkinson’s Disease clinical.

## Data Availability

The original contributions presented in the study are included in the article/Supplementary material, further inquiries can be directed to the corresponding author.
