# Supplementary material for: Evaluation of compliance and accuracy in Parkinson’s disease motor symptom tracking: a comparative study of digital and traditional paper diaries using a smartphone application (MyParkinson’s)
Source: Front Neurol. 2025 Jan 29;16:1522721. doi: 10.3389/fneur.2025.1522721 (PMC11814194; doi:10.3389/fneur.2025.1522721)
Supplement: Supplementary file 2 [file Data_Sheet_1.pdf]

Comparison of MyParkinson's and other Parkinson's Disease Motor Tracking Apps							
**Feature**	**My Parkinson's**	**mPower (Sage Bionetworks)**	**Parkinson mHealth (University of Rochester)**	**uMotif**	**Roche PD Mobile Application V2**	**Parkinson's Diary**	**OPDM (Ontario Parkinson Disease Management)**
**Real-time reporting**	Yes – Provides real-time logging and sharing of motor symptom data with clinicians for immediate monitoring and response.	No – Collects data but does not provide real-time access to clinicians.	No – Data is collected over time but not shared in real-time.	No – Data is collected and accessible by clinicians but not shared in real-time.	Yes – Offers real-time monitoring of motor symptoms and sends data to clinicians for timely review.	No – Logs motor symptoms daily but does not provide real-time data access.	Yes – Real-time data sharing with healthcare providers and clinicians for monitoring PD progression.
**Clinician interaction**	Yes – Direct integration with clinicians allowing them to receive real-time updates and review patient status remotely.	Limited – Mainly for research purposes with no direct clinical interaction features.	No – Focuses on self-managem ent and research with no clinician integration.	Yes – Allows data sharing with clinicians for review and long-term monitoring.	Yes – Clinicians can monitor symptoms remotely and review data in real-time.	No – Primarily for patient self-reporting with no clinician interface.	Yes – Clinician access to real-time data for adjusting treatment and tracking patient progress.

**Symptom logging**	Motor symptoms – Tracks key PD motor symptoms (tremors, dyskinesia, on-off states). Plans to expand to non-motor symptoms in future updates.	Wide range – Includes both motor and non-motor symptoms along with daily tasks and walking assessments.	Motor symptoms – Limited to tremor and dyskinesia with fewer options for non-motor symptoms.	Wide range – Tracks motor and non-motor symptoms, medication adherence, and well-being through a customizable interface.	Motor and non-motor symptoms – Tracks tremors, dyskinesia, bradykinesia, along with non-motor symptoms like sleep and mood changes.	Motor symptoms – Focuses on tremor, dyskinesia, and freezing episodes.	Tracks motor and non-motor symptoms, cognitive changes, and medication effectiveness.
**Reminders for entries**	Yes – Sends regular prompts to ensure patients log their symptoms on time. No backtracking allowed to maintain data accuracy.	Yes – Reminds users to perform specific tasks but allows for delayed entry.	Yes – Prompts patients but allows for more flexible entry timing which can introduce recall bias.	Yes – Sends reminders to track symptoms and medication adherence at regular intervals.	Yes – Sends alerts and reminders for symptom tracking and medication schedules.	Yes – Allows manual reminder setup but no automated prompts.	Yes – Automated prompts for symptom logging, medication adherence, and cognitive testing.
**Multilingual support**	Under development – Currently available in Turkish with plans for English and other languages.	No – Only available in English, limiting accessibility for non-English-speaking patients.	No – Available only in English.	Yes – Available in multiple languages including English, French, and Spanish.	Yes – Available in multiple languages, including English, French, Spanish, and German.	No – English only.	No – English and French available.

**Cost**	Free – Available without any subscription or in-app purchases.	Free – Funded through research grants making it freely available to users.	Free – Supported by research but may require institutional access.	Free – Available to patients participating in studies, or as part of clinical partnerships.	Free – Provided to clinical trial participants and may be commercially available in future.	Free – Open access to patients and caregivers.	Free – Funded by the Ontario government and available to residents as part of their care plan.
**Customization**	Yes – Personalized tracking based on patient preferences and clinician instructions.	Limited – Tasks are pre-determine d and cannot be customized to the individual's treatment plan.	No – Users follow a set protocol with no room for customization.	Yes – Highly customizable interface allowing patients to log personalized symptoms and activities.	Yes – Customizabl e based on patient needs and allows for personalized feedback from clinicians.	Limited – Pre-set symptom tracking with minor customization.	Yes – Highly customizable, allows tracking based on personal and clinician feedback.
**Data sharing**	Yes – Patients can share real-time reports with their physicians through a unique access code.	Yes – Data can be shared with researchers but not directly with personal clinicians.	Yes – Data can be shared for research purposes but lacks a direct physician connection.	Yes – Data can be shared with clinicians and researchers for personalized care and study analysis.	Yes – Real-time data sharing with clinicians is supported for real-time adjustments and clinical review.	No – Data remains on the app without external sharing functionality.	Yes – Data shared with clinicians for ongoing treatment adjustment and care management.
**User interface**	Simple and accessible – Designed for ease of use particularly for older patients with motor impairments.	Moderately complex – Includes multiple tasks which can be overwhelming for some users.	Basic – Limited functionality and not optimized for older users or those with advanced PD.	Simple and intuitive – Focuses on ease of use, with customizable options for symptom tracking.	Simple and accessible – Designed with a focus on ease of use and accessibility for PD patients.	Basic – Designed for quick symptom logging with minimal features.	Moderate – Designed for a broad range of tasks including symptom tracking, cognitive assessments,

							and caregiver feedback.
**Feedback**	Immediate – Patients and clinicians receive immediate visual feedback about symptoms.	Delayed – Users may review their data but real-time feedback is not provided.	No feedback – Patients record data but the app does not provide any form of instant feedback.	Immediate – Users can see immediate feedback on their entries; clinicians can review data trends over time.	Immediate – Both patients and clinicians receive real-time feedback based on symptom tracking.	No – Data is stored without providing instant feedback to the patient.	Immediate – Provides personalized feedback based on symptom logging and trends over time.
**Special Features**	Clinician dashboard – Clinicians can track patient progress and make adjustments based on real-time data.	Task-based assessments – Includes cognitive and walking tasks to assess daily functioning.	Limited – Primarily focuses on tremor and medication timing.	Comprehensive tracking – Supports long-term studies and personalized care with a focus on both motor and non-motor symptoms.	Advanced algorithms – Uses machine learning algorithms to analyze symptom progression and offers personalized insights for clinical decisions.	Symptom diary – Simple logging of motor fluctuations, medication adherence, and tremors.	Comprehensive platform – Tracks a wide range of symptoms and includes cognitive assessments, patient education resources, and direct links to caregivers and clinicia
